# Acute Effects of Marathon and Ultramarathon Running on Body Composition in Trained Male Athletes

**DOI:** 10.3390/medicina61122123

**Published:** 2025-11-28

**Authors:** Serkan Düz, İsmail İlbak, Ayşe Eda Kınacı Öğüt, Peter Sagat, Peter Bartik

**Affiliations:** 1 Department of Coaching Education, Faculty of Sport Sciences, İnönü University, Malatya 44050, Türkiye; serkan.duz@inonu.edu.tr; 2 Institute of Health Sciences, İnönü University, Malatya 44050, Türkiye; kinaciayseeda@gmail.com; 3 Sport Sciences and Diagnostics Research Group, College of Sciences and Humanities, Prince Sultan University, Riyadh 11586, Saudi Arabia; sagat@psu.edu.sa (P.S.); pbartik@psu.edu.sa (P.B.)

**Keywords:** body composition, marathon, ultramarathon, hydration, fat-free mass, bioelectrical impedance analysis

## Abstract

*Background and Objectives:* This study aimed to compare acute changes in body composition parameters following marathon (42.195 km) and ultramarathon (61 km) runs in trained male athletes, with particular focus on hydration dynamics and metabolic stress. *Materials and Methods:* Sixteen male amateur endurance runners were assigned to two groups: marathon (*n* = 8) and ultramarathon (*n* = 8). Body composition was assessed at three time points pre-race, immediately post-race, and 24 h post-race using bioelectrical impedance analysis. Measurements included body weight (BW), body mass index (BMI), total body water (TBW), total body fat (TBF), lean body mass (LBM), right arm fat (RAF), left arm fat (LAF), right leg fat (RLF), left leg fat (LLF), and torso fat (TF). *Results:* Both groups exhibited significant reductions in BW and BMI post-race (*p* < 0.05), with more pronounced changes observed in the ultramarathon group. Partial restoration of these metrics occurred within 24 h, primarily due to glycogen resynthesis and fluid retention. TBW remained stable immediately post-race but increased notably during recovery, particularly in ultramarathon runners, suggesting more effective hydration responses. Muscle and fat-free mass changes were minimal but more favorable in the ultramarathon group. Both total and regional fat percentages declined significantly post-race in both groups, with ultramarathon runners showing greater reductions. *Conclusions:* Endurance running induces short-term but substantial alterations in body composition, with ultramarathon participation eliciting more pronounced metabolic and fluid balance responses. These findings highlight the importance of race-specific nutritional and hydration strategies tailored to event type and duration.

## 1. Introduction

Marathon running traditionally covers a distance of 42.195 km, whereas ultramarathon events extend beyond this distance, typically beginning at 50 km [1,2]. While both disciplines fall under the category of endurance running, research has highlighted notable differences in training methodologies, physiological adaptations, and anthropometric profiles between marathon and ultramarathon runners [3].

A systematic review by Knechtle and Nikolaidis [3] highlighted that marathon runners generally exhibit higher total body fat (TBF) percentages, greater skinfold thickness, and larger limb girths compared to their ultramarathon counterparts. Specifically, marathoners tend to have smaller calf circumferences but thicker upper body skinfolds, whereas ultramarathon runners are characterized by leaner upper limbs and thighs, along with lower skinfold values across the torso [4,5]. These morphological differences are largely attributed to the distinct training regimens followed by each group.

Training volume and intensity serve as key distinguishing factors between marathon and ultramarathon runners. While marathon athletes typically emphasize pace and threshold intensities, ultramarathon runners prioritize duration and total weekly mileage, often training at lower intensities but for extended periods [6,7,8]. Consequently, each group encounters distinct physiological demands, which influence energy expenditure patterns, substrate utilization, and fluid requirements. These differences also have important implications for race-day strategies and post-race recovery protocols.

From a metabolic perspective, endurance athletes face two primary challenges: energy deficit and the risk of exercise-associated hyponatremia (EAH) due to excessive fluid intake [3]. Energy deficit occurs when energy expenditure surpasses intake during prolonged exertion, potentially impairing performance, accelerating muscle breakdown, and delaying recovery [9,10]. Conversely, overhydration can dilute serum sodium concentrations, leading to EAH—a condition that ranges from mild cognitive disturbances to severe neurological complications, including seizures and coma [11,12].

Given the physiological stressors associated with endurance events, the regulation of energy intake and hydration is crucial for maintaining performance and ensuring athlete safety both during and after competition [13]. Recent literature increasingly emphasizes the role of body composition analysis as a diagnostic tool for monitoring training load, fatigue, and recovery status [14,15,16,17]. In this context, key parameters such as body mass, lean mass, body fat percentage, and total body water may be effective in providing valuable information about the acute physiological responses to prolonged exercise.

While numerous studies have examined the anthropometric and body composition profiles of marathon and ultramarathon runners, empirical data on acute changes in response to competition remains limited. In particular, the dynamics of body composition from pre-race to immediate post-race and up to 24 h post-race are not well understood. Therefore, this study aims to evaluate and compare short-term changes in body composition among marathon and ultramarathon runners by analyzing measurements at three critical time points: pre-race, immediately post-race, and 24 h post-race. We hypothesize that ultramarathon runners will exhibit greater fluctuations in body composition particularly in total body mass, water balance, and fat-free mass due to the extended duration and cumulative metabolic demands of ultra-endurance racing.

## 2. Materials and Methods

### 2.1. Participants

The required sample size was estimated using G*Power version 3.1.9.7 (University of Düsseldorf, Düsseldorf, Germany) to ensure adequate statistical power for the study design. A repeated-measures ANOVA (within-between interaction) was selected as the analytical model. The parameters set for the calculation included an alpha level of 0.05, a minimum effect size (f) of 0.35, two independent groups, three repeated measurements, and a desired power of 0.80. The analysis indicated that a minimum of 16 participants would be sufficient to achieve approximately 83% statistical power. Accordingly, sixteen trained male endurance runners voluntarily participated in the study. Participants were allocated to the marathon (*n* = 8) and ultramarathon (*n* = 8) groups according to their training background and recent competition experience. Inclusion criteria required participants to be between 35–55 years of age and to have a minimum of three years of continuous endurance training, along with recent participation in events corresponding to their respective race categories. These criteria were implemented to ensure that all athletes possessed an established and comparable level of running proficiency. All participants were healthy, free from injury, and provided written informed consent prior to enrollment. Individuals who used alcohol or tobacco, had any neurological, psychological, or metabolic disease, or had a history of traumatic brain injury were excluded from the study. Descriptive characteristics for each group are summarized in Table 1.

### 2.2. Study Design

**Figure 1:**
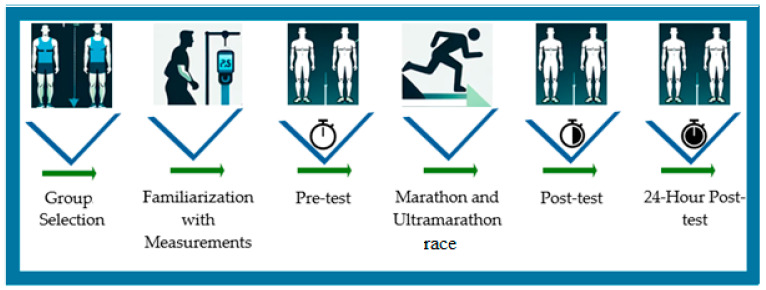
Schematic representation of the experimental design.

This cross-sectional study included 16 trained male athletes who were allocated into two groups: marathon (*n* = 8) and ultramarathon (*n* = 8). To accurately evaluate the acute effects of endurance running on body composition, measurements were performed at clearly defined time points. The baseline assessment was conducted approximately 30 min before the race start, following a standardized pre-race preparation routine. The second measurement was obtained immediately after athletes crossed the finish line (within the first 10 min) to capture acute exercise-induced alterations without delay. The final assessment was carried out exactly 24 h after race completion to evaluate short-term recovery-related changes. Body composition variables including body weight (BW), body mass index (BMI), total body water (TBW), total body fat (TBF), lean body mass (LBM), right arm fat (RAF), left arm fat (LAF), right leg fat (RLF), left leg fat (LLF), and torso fat (TF) were measured using a standardized protocol under identical environmental and procedural conditions across all time points. The collected data were analyzed to assess intra-group changes over time and inter-group differences between marathon and ultramarathon runners. The study protocol was reviewed and approved by the Inonu University Scientific Research and Publication Ethics Committee on 11 September 2024 (Session No: 14; Decision No: 2024/6379), and all procedures adhered to the ethical principles outlined in the Declaration of Helsinki. A schematic overview of the experimental design is presented in Figure 1.

### 2.3. Marathon and Ultramarathon Courses

#### Race Characteristics

**Figure 2:**
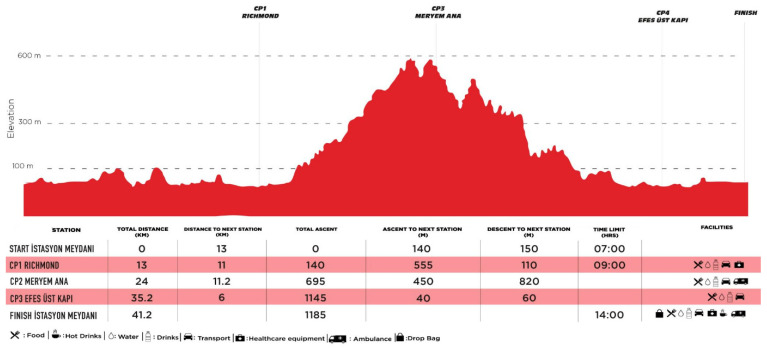
Schematic representation of marathon route.

The marathon and ultramarathon events examined in this study are part of the Efes Ultra Marathon, an internationally recognized trail running competition to be held in Türkiye in April 2025. The marathon course covered a 42 km route with a total elevation gain of 1185 m and a cut-off time of 7 h. The race commenced at 07:00 and concluded by 14:00 at the latest, with participants passing through three designated checkpoints (CP) along the route. The specific details of the racecourse are illustrated in Figure 2. Athletes were allowed to consume food, water, and hypotonic drinks at CP that had to be reached within certain time limits. All marathon participants successfully completed the race within the designated time limit.

**Figure 3:**
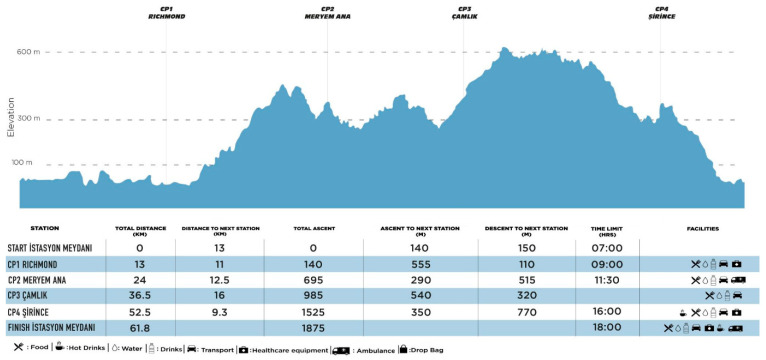
Schematic representation of ultramarathon route.

The ultramarathon course spanned 61 km, featuring a total elevation gain of 1875 m and a cut-off time of 11 h. The race commenced at 07:00 and concluded by 18:00 at the latest, with participants passing through three designated checkpoints (CP) along the route. The specific details of the racecourse are illustrated in Figure 3. Athletes were allowed to consume food, water, and hypotonic drinks at CP that had to be reached within certain time limits. All ultramarathon participants successfully completed the race within the designated time limit.

### 2.4. Body Composition Measurements

Body composition analyses were performed using the InBody 270 device (Biospace, Seoul, Republic of Korea), a bioelectrical impedance analysis system validated for multi-segmental body composition assessment. Prior to the measurements, a brief familiarization session was conducted to ensure that participants adopted the correct posture, maintained stable electrode contact, and achieved consistency across repeated assessments. All measurements were conducted with participants wearing only shorts; T-shirts, footwear, accessories, and any items that could add additional weight were removed. After the race, to prevent moisture-related measurement variability, athletes were not provided with dry clothing; instead, visible perspiration on the skin surface was carefully removed using a towel before the assessment. This standardized procedure was applied at all time points. During each measurement, participants stood barefoot on the platform, ensuring proper electrode contact through both the foot and hand electrodes. They were instructed to fully extend their elbows, maintain their shoulders at approximately a 30-degree angle, and remain still for 30 s. The device provided multiple body composition parameters, including body weight (kg), body mass index (kg/m^2^), total body water (%), total body fat (%), lean body mass (kg), and segmental fat distribution in the right arm, left arm, right leg, left leg, and trunk (%). BMI was included solely as a descriptive anthropometric variable, as it reflects overall mass but does not differentiate between changes in fat, muscle, or water compartments. Therefore, all interpretations of body composition changes were based on the compartment-specific BIA-derived variables rather than BMI.

### 2.5. Statistical Analysis

All statistical analyses were performed using IBM SPSS Statistics software (version 26.0; Armonk, NY, USA). Data normality was assessed through Skewness and Kurtosis values, ensuring they remained within the acceptable range of ±2, confirming a normal distribution. To examine the effects of race type (marathon vs. ultramarathon) on participants’ body composition over time, a Mixed Analysis of Variance (Mixed ANOVA) with a 2 × 3 design (group × time) was utilized. The dependent variables encompassed various body composition metrics, while the independent variables included group (between-subjects factor) and time (within-subjects factor).

Post hoc comparisons were conducted to identify specific pairwise differences among time points and between groups when significant main or interaction effects were detected. Homogeneity of variance assumptions were evaluated using Levene’s Test and Box’s M Test (*p* > 0.05), with Wilks’ Lambda or Hotelling’s Trace values reported accordingly. Sphericity was assessed using Mauchly’s Test of Sphericity, and when violated, Greenhouse-Geisser corrections were applied; otherwise, Sphericity Assumed results were reported. The statistical significance threshold for all analyses was set at *p* < 0.05.

## 3. Results

The results of this study are presented in the tables below. The dependent variables include BW, BMI, TBW, TBM, BF, LBM, RAF, LAF, RLF, LLF, and TF. The independent variables encompass Pre-test, Post-test, and 24-h Post-test measurements.

An examination of Table 2 indicates that in the marathon group, BMI decreased by 1.65% from pre-test to post-test, followed by a partial recovery of 1.22% after 24 h. In the ultramarathon group, a more pronounced reduction of 3.46% was observed post-test, with a subsequent recovery of 2.68% after 24 h. Regarding BW, the marathon group experienced a 1.76% decrease from pre-test to post-test, followed by a recovery of 1.37% after 24 h. In contrast, the ultramarathon group exhibited a notable weight reduction of 3.39% post-test, with a recovery of 2.53% recorded after 24 h.

In terms of TBW percentage, the marathon group showed a 0.40% increase from pre-test to post-test, which further increased to 1.19% after 24 h. In the ultramarathon group, a minimal increase of 0.09% was recorded post-test, followed by a more significant increase of 2.20% after 24 h.

Regarding TBM, the marathon group demonstrated a modest increase of 0.88% from pre-test to post-test, which further rose to 1.33% after 24 h. In contrast, the ultramarathon group showed a 0.49% increase post-test, with a more pronounced gain of 2.43% recorded after 24 h.

Concerning TBF percentage, the marathon group exhibited a reduction of 9.54% from pre-test to post-test, followed by a partial recovery to 6.73% after 24 h. In contrast, the ultramarathon group experienced a more pronounced decrease of 15.32% post-test, with a subsequent recovery to 12.61% after 24 h.

LBM in the marathon group increased by 0.52% from pre-test to post-test, with a further rise to 1.23% after 24 h. In the ultramarathon group, a negligible change of 0.02% was observed post-test, followed by a more substantial increase of 2.20% after 24 h.

Regional fat distribution exhibited reductions in both right and left arm and leg fat percentages across both groups from pre-test to post-test. In the marathon group, these reductions ranged between 10–12%, whereas in the ultramarathon group, the decreases were more pronounced, varying between 15–20%. Regarding TF percentage, both groups experienced reductions. The marathon group showed an 8.93% decrease, while the ultramarathon group exhibited a more substantial reduction of 17.14%.

Table 3 presents the effects of time and time × group interactions on the dependent variables. A statistically significant difference was observed across all dependent variables over time (*p* < 0.01). However, the time × group interactions did not yield a statistically significant effect (*p* > 0.05).

Table 4 illustrates a significant decrease in BMI from pre-test to post-test in both the marathon *p* < 0.01) and ultramarathon (*p* < 0.01) groups. Between the post-test and 24-h post-test, a partial recovery was observed, with a statistically significant increase in BMI (*p* < 0.05; *p* < 0.01, respectively). However, no significant difference was detected between the pre-test and 24-h post-test measurements.

With respect to BW, both the marathon and ultramarathon groups exhibited a significant decrease from pre-test to post-test (*p* < 0.01; *p* < 0.01). Between the post-test and 24-h post-test, a recovery was observed, accompanied by a statistically significant increase (*p* < 0.01; *p* < 0.01). However, no significant difference was detected between the pre-test and 24-h post-test measurements.

For TBW, no significant differences were observed between the pre-test and post-test or between the post-test and 24-h post-test in the marathon group. Similarly, in the ultramarathon group, no significant differences were detected between the pre-test and post-test or between the pre-test and 24-h post-test. However, a statistically significant increase in TBW was noted between the post-test and 24-h post-test (*p* < 0.05). In terms of TBM percentage, no statistically significant differences were detected between tests in either the marathon or ultramarathon groups.

As for LBM, no significant differences were observed between the pre-test and post-test or between the pre-test and 24-h post-test in either the marathon or ultramarathon groups. However, while no significant difference was detected between the post-test and 24-h post-test in the marathon group, the ultramarathon group exhibited an increase, and this difference was found to be statistically significant (*p* < 0.05).

Table 5 presents the findings on TBF percentage. No significant differences were observed between the pre-test and post-test or between the pre-test and 24-h post-test in either the marathon or ultramarathon groups. However, while no significant change was detected between the post-test and 24-h post-test in the marathon group, the ultramarathon group exhibited an increase, which was found to be statistically significant (*p* < 0.05).

For RAF, a significant decrease was observed between the pre-test and post-test in the marathon group (*p* < 0.01). Although a partial increase occurred between the post-test and 24-h post-test, it was not statistically significant. No significant difference was detected between the pre-test and 24-h post-test. Similarly in the ultramarathon group, significant differences in RAF were observed both between the pre-test and post-test and between the pre-test and 24-h post-test (*p* < 0.01; *p* < 0.05). While a partial increase was noted between the post-test and 24-h post-test, it did not reach statistical significance.

Regarding LAF, no significant differences were found in the marathon group across any test comparisons. However, in the ultramarathon group, significant differences were observed both between the pre-test and post-test and between the pre-test and 24-h post-test (*p* < 0.01; *p* < 0.05, respectively). A partial increase was recorded between the post-test and 24-h post-test, though it was not statistically significant.

For RLF, a statistically significant decrease was observed only between the pre-test and post-test in the marathon group (*p* < 0.01), while no significant differences were detected in the other comparisons. In the ultramarathon group, significant differences were found both between the pre-test and post-test and between the pre-test and 24-h post-test (*p* < 0.01; *p* < 0.05, respectively). Although a partial increase was noted between the post-test and 24-h post-test, it did not reach statistical significance. Similarly, for LLF, a statistically significant decrease was observed only between the pre-test and post-test in the marathon group (*p* < 0.01), while no significant differences were detected in the other comparisons. In the ultramarathon group, significant differences were found both between the pre-test and post-test and between the pre-test and 24-h post-test (*p* < 0.01; *p* < 0.05, respectively). However, no significant difference was observed between the post-test and 24-h post-test.

For the final parameter, TF, no significant differences were observed across any test comparisons in the marathon group. However, in the ultramarathon group, significant differences were detected both between the pre-test and post-test and between the pre-test and 24-h post-test (*p* < 0.01; *p* < 0.05, respectively). Although a partial increase was noted between the post-test and 24-h post-test, it did not reach statistical significance.

## 4. Discussion

This study aimed to investigate the short-term effects of marathon and ultramarathon running on body composition parameters in trained male athletes. Measurements were conducted at three time points pre-race, immediately post-race, and 24 h post-race to assess both the acute impact of endurance running and the early recovery dynamics. The findings largely support the initial hypothesis, indicating that ultramarathon runners experience more pronounced physiological alterations due to the prolonged metabolic and mechanical demands associated with ultra-endurance activity.

Both groups exhibited statistically significant reductions in BW and BMI from pre- to post-race, with the ultramarathon group experiencing more pronounced losses. These findings align with previous research indicating that increased energy expenditure during prolonged exercise promotes fat mobilization, leading to reductions in BW and BMI. Similar outcomes have been reported in endurance athletes by Bizjak et al. [18] and Rust et al. [4]. In the present study, BMI decreased by 1.65% in the marathon group and by 3.46% in the ultramarathon group. BW declined by 1.76% and 3.39%, respectively. Within 24 h, partial restoration of both variables was observed more prominently in the ultramarathon group likely due to glycogen resynthesis and fluid retention, a mechanism supported by Tiller et al. [19].

TBW levels showed minimal change immediately post-race; however, because fluid intake and hydration behaviors during the event were not recorded, no definitive conclusions can be drawn regarding the maintenance of hydration or electrolyte balance. Therefore, the observed reduction in body mass cannot be attributed solely to energy expenditure or interpreted as evidence of adequate hydration. Existing literature suggests that body mass loss during ultramarathon events is often associated with decreases in fat mass rather than dehydration [13,20], but the present findings should be interpreted cautiously given the absence of fluid-intake data. In the ultramarathon group, TBW increased by 2.20% at 24 h post-race, which may reflect post-race rehydration, although individual variability cannot be ruled out. This observation is consistent with studies reporting post-event increases in TBW following prolonged endurance exercise [20]. No hyponatremia-related complications were observed in the current sample; however, without detailed monitoring of hydration status, the potential risks of both dehydration and overhydration must be acknowledged [21].

No significant changes in LBM were observed across time points in either group, suggesting that muscle mass remained relatively stable during a single endurance event. Previous research indicates that muscle loss or damage in such events may vary depending on factors such as intensity, duration, and individual conditioning [3,22,23,24,25]. However, the absence of change in the present study should be interpreted cautiously, as detailed information on individual running experience, training load, and performance level was not collected. Given the inclusion criteria which required at least three years of continuous endurance training and recent participation in the respective race categories all participants likely possessed a comparable baseline conditioning level. Even so, more precise performance-related data would be needed to conclusively determine whether pre-race training status contributed to the stability of LBM.

LBM remained stable in the marathon group but increased by 2.20% in the ultramarathon group from post-race to 24 h. However, this change should be interpreted with caution, as the measurement technique and available data do not allow for conclusions regarding the underlying physiological mechanisms. Comparable trends have been reported in the literature [26], while other studies have documented decreases in lean mass following ultra-endurance events, often attributed to catabolic responses to energy deficits [18,27]. These inconsistencies highlight the challenges of interpreting body composition outcomes over short timeframes and emphasize the need for more detailed monitoring in future research.

Reductions in total and regional body fat percentages were more pronounced in the ultramarathon group. TBF decreased by 15.32% post-race, compared to 9.54% in the marathon group. Segmental fat loss (arms, legs, trunk) followed similar patterns, with greater decreases observed in the ultramarathon group. These findings reinforce the notion that longer-duration endurance events induce more significant metabolic stress. Supporting studies have demonstrated substantial fat loss in ultramarathon runners, including visceral fat reductions exceeding 60% during transcontinental races [27] and measurable fat mass loss in 100 km events [8]. Although direct comparisons between marathon and ultramarathon runners remain limited, existing evidence suggests that the prolonged duration and higher cumulative energy expenditure of ultramarathon events result in more pronounced fat loss. The current results align with Knechtle and Nikolaidis [3], who reported higher body fat and skinfold values in marathon runners compared to ultramarathoners.

These findings should be interpreted in light of several important limitations. The limited sample size (*n* = 8 per group) may constrain statistical power particularly given the small effect sizes typically observed in repeated-measures physiological data and therefore restrict the generalizability of the results. In addition, the exclusive inclusion of male athletes limits the ability to draw sex-based comparisons. Although body composition measurements were conducted using a standardized protocol, the accuracy of bioelectrical impedance analysis can be influenced by hydration fluctuations, gastrointestinal contents, and acute fluid shifts. Because fluid and nutritional intake during the race were not recorded, and because only a towel-drying procedure was used following heavy perspiration, some degree of variability in post-race and 24-h follow-up measurements cannot be ruled out. Environmental factors such as temperature and humidity were not controlled, which may have further influenced physiological responses and recovery dynamics. Moreover, individual target race times which could provide additional insight into participants’ performance level were not collected, limiting the ability to contextualize individual variation in physiological responses. Furthermore, the study focused solely on acute responses to endurance running and did not assess longer-term physiological adaptations.

Future research should incorporate larger and more diverse samples, including female athletes, to improve representativeness and enable sex-specific analyses. Standardized monitoring of environmental conditions, as well as controlled or recorded post-race hydration and nutrition behaviors, would enhance the interpretability of body composition outcomes. Collecting individualized performance-related variables such as target race times or training load may also help better contextualize physiological responses across athletes. Additionally, the inclusion of biochemical markers, hydration indices, and performance parameters would provide a more comprehensive understanding of the mechanisms underlying acute and longer-term changes in body composition following endurance events.

## 5. Conclusions

This study demonstrated that marathon and ultramarathon running lead to acute alterations in body composition, with ultramarathon participation eliciting more pronounced changes across several parameters. Both groups experienced significant reductions in body weight, BMI, and regional fat percentages immediately after the race, but the magnitude of these reductions was consistently greater in ultramarathon runners, reflecting the higher metabolic cost and prolonged mechanical demands of ultra-endurance activity. While TBW and other hydration-related variables exhibited partial recovery within 24 h, these shifts should be interpreted with caution, as fluid intake and hydration behaviors during the event were not recorded. Lean body mass remained largely stable across time points, suggesting that acute endurance exercise affected fluid and fat compartments more strongly than muscle tissue. Overall, the findings indicate that race distance is an important determinant of acute physiological stress and post-race recovery dynamics. These results highlight the value of race-specific recovery protocols particularly regarding hydration, electrolyte management, and nutritional strategies to support efficient restoration of energy balance and body composition after prolonged endurance exercise. Despite the insights obtained, the study’s small sample size, limited participant diversity, and absence of detailed hydration, nutritional, environmental, and performance-related data restrict broader generalization. Future studies incorporating larger and more heterogeneous cohorts, controlled assessment conditions, and complementary biochemical and performance indicators will be essential for advancing the understanding of both acute and longer-term adaptations to endurance running.

## Figures and Tables

**Table 1:** Descriptive characteristics of the participants.

Group		Minimum	Maximum	Mean	SD
Marathon (*n* = 8)	Age (year)	38.00	53.00	45.25	5.77
	Body Height (cm)	171.00	187.00	177.63	4.59
	Body Weight (kg)	66.40	99.20	80.31	11.39
Ultramarathon (*n* = 8)	Age (year)	38.00	50.00	43.00	4.47
	Body Hight (cm)	162.00	188.00	174.13	9.03
	Body Weight (kg)	59.90	90.20	74.70	11.06

**Table 2:** Mean Values of Dependent Variables by Time × Groups.

Dependent Variables (DV)	Independent Variables (IV)	Mean ± SD	
		Marathon	Ultramarathon
Body Mass Index (kg/m^2^)	Pre-test	25.45 ± 3.49	24.56 ± 2.28
	Post-test	25.02 ± 3.59	23.71 ± 2.32
	24 h Post-test	25.33 ± 3.60	24.33 ± 2.24
Total Body Weight (kg)	Pre-test	80.31 ± 11.39	74.70 ± 11.06
	Post-test	78.90 ± 11.53	72.16 ± 11.03
	24 h Post-test	79.98 ± 11.65	74.05 ± 10.61
Total Body Water (%)	Pre-test	47.46 ± 4.85	44.36 ± 5.63
	Post-test	47.65 ± 5.32	44.40 ± 5.95
	24 h Post-test	48.02 ± 5.01	45.33 ± 6.31
Total Body Muscle (%)	Pre-test	36.83 ± 3.99	34.32 ± 4.71
	Post-test	37.16 ± 4.41	34.50 ± 5.01
	24 h Post-test	37.32 ± 4.15	35.16 ± 5.32
Total Body Fat (%)	Pre-test	18.57 ± 7.86	18.40 ± 3.58
	Post-test	16.80 ± 7.52	15.58 ± 3.92
	24 h Post-test	17.32 ± 7.30	16.07 ± 3.39
Lean Body Mass (kg)	Pre-test	64.81 ± 6.54	60.73 ± 7.82
	Post-test	65.15 ± 7.21	60.72 ± 8.27
	24 h Post-test	65.61 ± 6.80	62.07 ± 8.79
Right Arm Fat (%)	Pre-test	0.85 ± 0.79	0.75 ± 0.24
	Post-test	0.68 ± 0.74	0.52 ± 0.23
	24 h Post-test	0.75 ± 0.75	0.58 ± 0.20
Left Arm Fat (%)	Pre-test	0.86 ± 0.79	0.77 ± 0.27
	Post-test	0.73 ± 0.70	0.55 ± 0.27
	24 h Post-test	0.77 ± 0.73	0.60 ± 0.20
Right Leg Fat (%)	Pre-test	2.32 ± 1.13	2.20 ± 0.54
	Post-test	1.96 ± 0.90	1.73 ± 0.48
	24 h Post-test	2.15 ± 1.10	1.88 ± 0.41
Left Leg Fat (%)	Pre-test	2.32 ± 1.14	2.18 ± 0.53
	Post-test	1.96 ± 0.90	1.72 ± 0.47
	24 h Post-test	2.12 ± 1.08	1.86 ± 0.42
Torso Fat (%)	Pre-test	7.97 ± 4.78	6.98 ± 2.53
	Post-test	7.26 ± 4.83	5.78 ± 2.58
	24 h Post-test	7.26 ± 4.76	6.01 ± 2.17

**Table 3:** ANOVA Test Results for Dependent Variable Effects by Time × Group Interaction.

Dependent Variables	Time	Sum of Squares	df	Mean Square	F	*p*	ηp^2^
Body Mass Index (kg/m^2^)	Time	3.49	2	1.74	37.19	0.01 **	0.72
	Time × Group	0.38	2	0.19	4.13	0.05	0.22
Total Body Weight (kg)	Time	33.87	2	16.93	33.05	0.01 **	0.70
	Time × Group	2.68	2	1.34	2.61	0.09	2.68
Total Body Water (%)	Time	5.51	2	2.75	5.30	0.01 *	0.27
	Time × Group	0.67	2	0.33	0.65	0.52	0.04
Total Body Muscle (%)	Time	3.58	2	1.79	5.58	0.01 **	0.28
	Time × Group	0.52	2	0.26	0.82	0.45	0.05
Total Body Fat (%)	Time	39.08	2	19.54	20.30	0.01 **	0.59
	Time × Group	1.80	2	0.90	0.93	0.40	0.06
Lean Body Mass (kg)	Time	10.61	2	5.30	5.43	0.01 **	0.28
	Time × Group	1.59	2	0.79	0.81	0.45	0.05
Right Arm Fat (%)	Time	0.31	2	0.15	21.54	0.01 **	0.60
	Time × Group	0.01	2	0.00	0.71	0.49	0.04
Left Arm Fat (%)	Time	0.26	2	0.13	16.09	0.01 **	0.53
	Time × Group	0.02	2	0.01	1.44	0.25	0.09
Right Leg Fat (%)	Time	1.37	2	0.68	20.57	0.01 **	0.59
	Time × Group	0.04	2	0.02	0.60	0.54	0.04
Left Leg Fat (%)	Time	1.39	2	0.69	20.70	0.01 **	0.59
	Time × Group	0.03	2	0.01	0.51	0.60	0.03
Torso Fat (%)	Time	8.74	2	4.37	12.74	0.01 **	0.47
	Time × Group	0.47	2	0.23	0.69	0.50	0.04

** *p* < 0.01; * *p* < 0.05; ηp^2^: partial eta squared.

**Table 4:** Post Hoc Test Results for BMI, BW, TBW, TBM and LBM.

Dependent Variables	Group	Time		Mean Difference	Std. Error	*p*
Body Mass Index (kg/m^2^)	Marathon	Pre-test	Post-test	−0.42	0.10	0.01 **
			24 h Post-test	−0.11	0.12	1.00
		Post-test	24 h Post-test	0.31	0.09	0.01 **
	Ultra-marathon	Pre-test	Post-test	−0.85	0.10	0.01 **
			24 h Post-test	−0.22	0.12	0.26
		Post-test	24 h Post-test	0.62	0.09	0.01 **
Total Body Weight (kg)	Marathon	Pre-test	Post-test	−1.41	0.37	0.01 **
			24 h Post-test	−0.32	0.39	1.00
		Post-test	24 h Post-test	1.08	0.29	0.01 **
	Ultra-marathon	Pre-test	Post-test	−2.53	0.37	0.01 **
			24 h Post-test	−0.65	0.39	0.37
		Post-test	24 h Post-test	1.88	0.29	0.01 **
Total Body Water (%)	Marathon	Pre-test	Post-test	0.18	0.33	1.00
			24 h Post-test	0.56	0.42	0.63
		Post-test	24 h Post-test	0.37	0.30	0.70
	Ultra-marathon	Pre-test	Post-test	0.03	0.33	1.00
			24 h Post-test	0.97	0.42	0.11
		Post-test	24 h Post-test	0.93	0.30	0.02 *
Total Body Muscle (%)	Marathon	Pre-test	Post-test	0.32	0.26	0.73
			24 h Post-test	0.48	0.33	0.48
		Post-test	24 h Post-test	0.16	0.24	1.00
	Ultra-marathon	Pre-test	Post-test	0.17	0.26	1.00
			24 h Post-test	0.83	0.33	0.07
		Post-test	24 h Post-test	0.66	0.24	0.05
Lean Body Mass (kg)	Marathon	Pre-test	Post-test	0.33	0.45	1.00
			24 h Post-test	0.80	0.58	0.57
		Post-test	24 h Post-test	0.46	0.42	0.88
	Ultra-marathon	Pre-test	Post-test	−0.01	0.45	1.00
			24 h Post-test	1.33	0.58	0.11
		Post-test	24 h Post-test	1.35	0.42	0.02 *

** *p* < 0.01; * *p* < 0.05.

**Table 5:** Post Hoc Test Results for Fat Distribution.

Dependent Variables	Group	Time		Mean Difference	Std. Error	*p*
Total Body Fat (%)	Marathon	Pre-test	Post-test	−0.33	0.45	1.00
			24 h Post-test	−0.80	0.58	0.57
		Post-test	24 h Post-test	0.46	0.42	0.88
	Ultra-marathon	Pre-test	Post-test	−0.01	0.45	1.00
			24 h Post-test	−1.33	0.58	0.11
		Post-test	24 h Post-test	1.35	0.42	0.02 *
Right Arm Fat (%)	Marathon	Pre-test	Post-test	−0.16	0.03	0.01 **
			24 h Post-test	−0.10	0.05	0.24
		Post-test	24 h Post-test	0.06	0.03	0.28
	Ultra-marathon	Pre-test	Post-test	−0.22	0.03	0.01 **
			24 h Post-test	−0.16	0.05	0.02 *
		Post-test	24 h Post-test	0.06	0.03	0.28
Left Arm Fat (%)	Marathon	Pre-test	Post-test	−0.12	0.04	0.05
			24 h Post-test	−0.08	0.05	0.31
		Post-test	24 h Post-test	0.03	0.03	1.00
	Ultra-marathon	Pre-test	Post-test	−0.22	0.04	0.01 **
			24 h Post-test	−0.17	0.05	0.01 *
		Post-test	24 h Post-test	0.05	0.03	0.61
Right Leg Fat (%)	Marathon	Pre-test	Post-test	−0.36	0.09	0.01 **
			24 h Post-test	−0.17	0.10	0.30
		Post-test	24 h Post-test	0.18	0.08	0.11
	Ultra-marathon	Pre-test	Post-test	−0.46	0.09	0.01 **
			24 h Post-test	−0.31	0.10	0.02 *
		Post-test	24 h Post-test	0.15	0.08	0.26
Left Leg Fat (%)	Marathon	Pre-test	Post-test	−0.36	0.09	0.01 **
			24 h Post-test	−0.20	0.10	0.20
		Post-test	24 h Post-test	0.16	0.07	0.15
	Ultra-marathon	Pre-test	Post-test	−0.46	0.09	0.01 **
			24 h Post-test	−0.32	0.10	0.01 *
		Post-test	24 h Post-test	0.13	0.07	0.28
Torso Fat (%)	Marathon	Pre-test	Post-test	−0.71	0.31	0.11
			24 h Post-test	−0.71	0.32	0.13
		Post-test	24 h Post-test	8.88	0.23	1.00
	Ultra-marathon	Pre-test	Post-test	−1.20	0.31	0.01 **
			24 h Post-test	−0.97	0.32	0.02 *
		Post-test	24 h Post-test	0.22	0.23	1.00

** *p* < 0.01; * *p* < 0.05.

## Data Availability

The original contributions presented in this study are included in the article. Further inquiries can be directed to the corresponding author.
